# Marketing digital de fórmulas comerciales infantiles en Argentina: un estudio etnográfico digital

**DOI:** 10.18294/sc.2024.4776

**Published:** 2024-04-23

**Authors:** Flavia Demonte, Daniela Paola Bruno, María Celeste Nessier, María Elisa Zapata

**Affiliations:** 1 Posdoctora en Ciencias Sociales. Investigadora Adjunta, Consejo Nacional de Investigaciones Científicas y Técnicas, con sede en Escuela Interdisciplinaria de Altos Estudios Sociales, Universidad Nacional de San Martín, Buenos Aires, Argentina. fdemonte@unsam.edu.ar Consejo Nacional de Investigaciones Científicas y Técnicas Consejo Nacional de Investigaciones Científicas y Técnicas Escuela Interdisciplinaria de Altos Estudios Sociales Universidad Nacional de San Martín Buenos Aires Argentina fdemonte@unsam.edu.ar; 2 Posdoctora en Ciencias Sociales. Investigadora, Instituto de Investigaciones Gino Germani, Facultad de Ciencias Sociales, Universidad de Buenos Aires. Investigadora, Facultad de Periodismo y Comunicación Social, Universidad Nacional de la Plata, Ciudad Autónoma de Buenos Aires, Argentina. danielapaolabruno@gmail.com Universidad Católica de La Plata Facultad de Periodismo y Comunicación Social Universidad Nacional de la Plata Ciudad Autónoma de Buenos Aires Argentina danielapaolabruno@gmail.com; 3 Magister en Ciencias de la Nutrición. Coordinadora de proyectos, Centro de Estudios en Nutrición Infantil “Dr. Alejandro O´Donnell”, Ciudad Autónoma de Buenos Aires, Argentina. Doctor en Ciencia de la Comunicación. Docente Investigador, Facultad de Arte, Comunicación y Cultura, Universitaria Agustiniana, Bogotá, Colombia. mcnessier@cesni.org.ar Universitaria Agustiniana Facultad de Arte, Comunicación y Cultura Universitaria Agustiniana Bogotá Colombia mcnessier@cesni.org.ar; 4 Doctora en Nutrición Humana. Directora, Centro de Estudios de Nutrición Infantil “Dr. Alejandro O´Donnell”, Ciudad Autónoma de Buenos Aires, Argentina. mezapata@cesni.org.ar Centro de Estudios de Nutrición Infantil Dr. Alejandro O´Donnell Centro de Estudios de Nutrición Infantil “Dr. Alejandro O´Donnell” Ciudad Autónoma de Buenos Aires Argentina mezapata@cesni.org.ar

**Keywords:** Sustitutos de la Leche Humana, Fórmulas Infantiles, Marketing, Investigación Cualitativa, Red Social, Argentina, Breast-Milk Substitutes, Infant Formula, Marketing, Qualitative Research, Social Networking, Argentina

## Abstract

Si bien es extendida la evidencia de los beneficios de la lactancia materna, diversos son los desafíos para iniciarla y sostenerla. Las empresas productoras de fórmulas infantiles utilizan estrategias de marketing violatorias de las regulaciones existentes, contribuyendo a su temprano abandono. Exploramos la exposición al marketing digital de las fórmulas infantiles en Argentina mediante el análisis de las interacciones de la población con las marcas y las huellas de dichas interacciones en conversaciones entabladas en grupos de Facebook durante 2022, desde un enfoque cualitativo basado en la etnografía digital. Los resultados muestran que las empresas despliegan tácticas elusivas de las regulaciones y buscan el contacto con las madres. Las usuarias no interactúan con las cuentas, pero están expuestas a sus estrategias dado el correlato entre los atributos del producto presentes en la publicidad con sus motivaciones y aspiraciones. Los mediadores entre el marketing y las madres son los profesionales médicos, utilizados como recursos del marketing. Concluimos que las autoridades deben promover nuevos acuerdos sobre las prácticas de los profesionales médicos y desarrollar regulaciones teniendo en cuenta los entornos digitales.

## Abstract (en)

Although evidence of the benefits of breastfeeding is widespread, there are several challenges to initiate and sustain it. Infant formula companies use marketing strategies that violate existing regulations, contributing to its early abandonment. We explore the digital marketing exposure of infant formulas in Argentina by analyzing people’s interactions with brands and the traces of these interactions in conversations engaged in Facebook groups during 2022, from a qualitative approach based on digital ethnography. Results show that companies deploy regulatory avoidance tactics and seek contact with mothers. Users do not interact with the accounts but are exposed to their strategies given the correlation between product attributes present in advertising with their motivations and aspirations. The mediators between marketing and mothers are medical professionals, used as marketing resources. We conclude that authorities should promote new agreements on the practices of medical professionals and develop regulations taking into account digital environments.

## INTRODUCCIÓN

Si bien es extendida la evidencia de los beneficios de la leche humana (LH)^1^^,^^2^^,^^3^ y a pesar de que organismos como la Organización Mundial de la Salud (OMS) y el Fondo de las Naciones Unidas para la Infancia (UNICEF) elaboraron directrices orientadas a promover la lactancia exclusiva los primeros seis meses del bebé, seguida de la incorporación de alimentos complementarios y lactancia hasta los dos años de edad y más^4^^,^^5^^,^^6^, determinantes económicos, comerciales, laborales, socioculturales e individuales plantean desafíos para iniciarla y sostenerla^7^^,^^8^^,^^9^^,^^10^. Diversas investigaciones identificaron barreras estructurales que socavan la lactancia, entre las que se destacan los mercados laborales que no se adaptan adecuadamente a los derechos reproductivos de las mujeres y al trabajo de cuidados generando desigualdades de género; las normas socioculturales sobre maternidad, cuidado y alimentación infantil, las prácticas sanitarias deficientes (incluida la medicalización del parto y del cuidado infantil), y las estrategias de marketing que debilitan las políticas de protección de la lactancia^2^^,^^3^^,^^10^. 

El estudio de los determinantes y de las barreras estructurales implica considerar a la lactancia y a la maternidad no solo como acontecimientos biológicos, sino también como fenómenos socioculturales^2^^,^^3^^,^^11^^)^ inscriptos en el trabajo de cuidados^10^ y asociados con significados, valores y normas a partir de inscripciones simbólicas y de construcciones sociohistóricas que le dan sentido a las prácticas y a las experiencias de los sujetos^12^. La lactancia es asumida como una práctica de cuidado y como alimentación infantil considerada saludable desde las políticas protectoras de la lactancia materna, pero también en ella están implicadas las experiencias corporales de las mujeres, el aprendizaje en la gestión de las emociones, las expectativas sociales en torno a la crianza y a la maternidad^13^^,^^14^. Así, las decisiones que se toman son el resultado de posibilidades, emociones, aprendizajes, experiencias y deben analizarse tomando en consideración los discursos normativos que marcan lo que una madre debe o no debe ser y hacer como producto de la atribución de roles de género diferenciados^9^^,^^12^^,^^15^^,^^16^. Por tanto, como fenómeno construido socialmente, conlleva procesos de negociación, resignificación y redefinición de identidades de género^12^, la presencia de tensiones y contradicciones ante las expectativas sociales que marcan un vínculo causal entre lactancia y “buena maternidad”^9^^,^^16^ y la reconfiguración de conocimientos y experiencias sobre la maternidad y los cuidados. Enmarcada como una cuestión de responsabilidad individual y, en particular, como responsabilidad exclusiva de las mujeres vinculada con el cuidado infantil^2^^,^^17^; experimentada en ocasiones como imperativo de salud pública o mandato social, cultural y moral^9^^,^^11^^,^^13^^,^^17^; demandada en otras como un derecho de las mujeres a decidir si practicarla o no^15^, la decisión de iniciar, suspender o continuar la lactancia implica reconocer y evaluar el deseo y la capacidad de las mujeres^12^^,^^15^, y especialmente las barreras estructurales que socavan su práctica como responsabilidad colectiva^2^^,^^3^. 

Una de las barreras estructurales son las estrategias de marketing de las empresas de sucedáneos de leche materna, productos que se presentan como sustitutos de la leche humana para niños de hasta dos o tres años (según el país)^5^^,^^18^^,^^19^^,^^20^, entre ellos, las fórmulas infantiles. Las estrategias de marketing de las fórmulas se nutren de los significados, las emociones, las experiencias y los valores sobre maternidad y lactancia y los aprovechan como argumentos de venta de las fórmulas. Lupton^17^ señala que el marketing y la publicidad buscan que las personas potencialmente consumidoras compren productos y los incorporen a su vida cotidiana. Para lograrlo, deben unir el producto a una representación del mundo culturalmente constituido, es decir, asociarlo con símbolos y valores conocidos. En términos de segmentación, buscan llegar a embarazadas -consideradas el “santo grial” para las ventas de fórmulas^21^-, mediante la utilización de algoritmos basados ​​en la recolección y sistematización de datos que dirigen la publicidad digital a mujeres cuyo comportamiento en línea sugiere que podrían estar embarazadas^22^.

La promoción de fórmulas no es novedosa^2^^,^^6^^,^^10^ y al menos desde 1974 existen evidencias que su publicidad influye en su uso^23^, desalentando la lactancia materna y contribuyendo a su temprano abandono^2^. Diversas investigaciones evidenciaron la utilización de estrategias de marketing atractivas y emotivas^19^^,^^20^^,^^24^^,^^25^ que enfatizan los productos por encima de su valor de uso real y los asocian con determinados significados, valores y estilos de vida^17^. En sus estrategias, presentan las fórmulas con efectos similares a la leche humana, argumentando sobre propiedades nutricionales avaladas cientificamente^8^^,^^24^, con capacidad para calmar a bebés irritables^3^ y como solución a los desafíos relacionados con el comportamiento y el cuidado infantil; como una elección de estilo de vida urbano, asociado con la movilidad social ascendente, que ubica a la mujer libre de condicionamientos biológicos, permitiendo su plena incorporación al mercado laboral y la participación masculina en la alimentación del lactante^2^ o como un derecho que tienen las madres de elegir lo mejor para sus hijos e hijas según sus circunstancias personales o laborales^7^. También las investigaciones muestran el rol de los profesionales de la salud^2^^,^^5^^,^^19^^,^^20^^,^^26^^)^ quienes difunden mensajes acerca de problemas de la lactancia que se resuelven con fórmulas, reforzando la medicalización de la alimentación y de los comportamientos infantiles^3^^,^^27^. 

Con la finalidad de regular la promoción inadecuada, en 1981 la OMS promovió el Código Internacional de Comercialización de Sucedáneos de Leche Materna (CICSLM)^22^, que prohíbe su promoción por medios de comunicación, obsequios a trabajadores y trabajadoras de la salud y madres, uso de declaraciones de propiedades saludables y promoción cruzada (*cross promotion*) con productos de alimentación complementaria; donaciones a servicios de salud y otros incentivos^2^^,^^7^^,^^18^. A pesar de los esfuerzos, la promoción se extendió a través de un marketing digital más interactivo, personalizado y encubierto^22^^,^^26^ ayudado por la tecnología digital, la recolección de datos personales, la inteligencia artificial^2^ y los procesos de digitalización de la vida cotidiana^28^. Diversas investigaciones identificaron las modalidades violatorias del CICSLM y evidenciaron el aumento de estas en el entorno virtual^18^^,^^19^^,^^24^. 

Si bien Argentina incorporó el CICSLM a su normativa, lo hizo parcialmente^29^. Investigaciones locales mostraron violaciones, por fuera del ecosistema digital, a través de donaciones de productos a instituciones de salud, entregas de muestras y regalos a madres y trabajadores y trabajadoras de la salud, contacto con madres, violaciones en el etiquetado y puntos de venta^30^. En esta línea, otras investigaciones mostraron cómo el marketing de sucedáneos de leche materna aprovecha medios de comunicación especializados, como las revistas médicas científicas, destinados a profesionales para realizar publicidad y promover la indicación de las fórmulas^6^. En estas reconstruyeron simbolizaciones de las fórmulas que, mediante lenguaje científico, enfatizan en sus propiedades nutricionales, idealizan su contribución al crecimiento infantil, y los asocian con el cuidado que deben demostrar padres y madres hacia la alimentación de sus hijos e hijas^29^. Así, los desafíos que expresan las mujeres sobre la lactancia^9^^,^^11^ a menudo coinciden con los planteados por las estrategias de marketing^5^. Además, las estrategias se basan en tácticas elusivas^2^, como promociones cruzadas entre productos de la misma empresa; iniciativas para alentar la búsqueda de asesoramiento; acceso a *carelines* (en español líneas de cuidado que, como táctica de marketing, refiere a asesorías o consejerías *on line* sobre temas de crianza y desarrollo infantil que son ofrecidas por las empresas); invitaciones a clubes de madres y padres con regalos y descuentos; interacción directa con la empresa a través del contacto *online*, presentándose como fuentes de confianza generadoras de apoyo para la crianza con profesionales médicos aliados^22^^,^^26^, socios o intermediarios^2^^,^^6^. Estas estrategias se implementan con la ayuda de herramientas provistas por los entornos digitales: cuentas en redes sociales, aplicaciones móviles, blogs, webs^31^^,^^32^ que difieren de las de la publicidad en medios masivos tradicionales o en revistas especializadas^6^.

Este estudio forma parte de una investigación mayor realizada en Argentina durante 2022-2023, que tuvo como objetivo describir las estrategias de marketing digital de sucedáneos de leche materna e identificar sus posibles efectos y violaciones al CICSLM, con el propósito de proporcionar recomendaciones de política pública^33^. A diferencia de la mayoría de los antecedentes que buscan identificar cómo se viola el CICSLM en entornos digitales, este estudio en particular se propuso como objetivo describir y analizar las interacciones de la población con las estrategias de marketing digital de fórmulas comerciales infantiles y las huellas de estas en las conversaciones entabladas entre la población usuaria de una red social en el propio entorno donde se despliegan las estrategias y las tácticas. 

Teniendo en cuenta el objetivo propuesto, se apeló a un abordaje interdisciplinario con enfoque interpretativo que recupera aportes de los estudios socio antropológicos de la salud, la alimentación y la nutrición; de los estudios sobre maternidad y crianza desde la perspectiva de géneros y, fundamentalmente, de los estudios de comunicación y análisis de la publicidad desde perspectivas teóricas que reconocen la naturaleza socialmente construida de los fenómenos desde un énfasis crítico que tiene en cuenta los contextos históricos, sociales, políticos y económicos en los que se producen y reproducen los conocimientos, significados, valores y normas^17^^,^^34^. Estas construcciones simbólicas pueden circular por diversos medios, lenguajes y formatos. Por tanto, las perspectivas de los actores de interés en este estudio (empresas y madres) pueden ser capturadas a través de posteos e intercambios discursivos en redes sociales digitales. 

## METODOLOGÍA

En este estudio se utilizó un enfoque cualitativo de carácter exploratorio basado en la utilización de herramientas de la etnografía digital, por su potencial heurístico para comprender la perspectiva de los actores sobre fórmulas infantiles a partir de interacciones y conversaciones en redes sociales digitales^35^. Se seleccionó Facebook, por varios criterios:

1. Es una de las redes más utilizadas en Argentina (al menos el 72% de la población mayor de 13 años tiene una cuenta)^36^.
2. Si bien la utiliza sobre todo la población mayor de 35 años, otras investigaciones^26^ eligieron Facebook porque las personas la utilizan para buscar información y consejos e interactuar en grupos sobre temas de interés. Los grupos son adecuados para comprender las perspectivas de los actores, ya que permiten observar qué temas interesan a las personas usuarias, cómo los expresan, consultan a otras y otros integrantes, se ayudan y responden entre sí^37^.
3. Como parte de sus estrategias de marketing digital las empresas pautan (pagan) posteos para animar la participación y con ello la recolección de datos de las personas usuarias. El análisis de las interacciones con las cuentas de las marcas en diálogo con el análisis de las conversaciones en los grupos de crianza resulta útil para controlar la posible distorsión de interacciones pautadas y reconstruir las huellas del marketing digital en las conversaciones de las personas usuarias.
4. Otras investigaciones destacan su potencial para el marketing multinivel: las empresas promocionan sus productos y alientan a usuarios a interactuar para que aporten datos y generar *insights* y elaborar perfiles sobre intereses y comportamientos del consumidor. Facebook recolecta datos que vende a terceros para facilitar publicidad en su plataforma^31^.

Con el fin de identificar las estrategias del marketing digital, se recolectaron y describieron posteos publicados en 2022 y las reacciones de usuarios de cuentas de las empresas de fórmulas infantiles más importantes de Argentina. Para identificar las huellas del marketing digital en las conversaciones de pares se recolectaron y describieron posteos y reacciones sobre fórmulas y lactancia materna en grupos de orientación sobre maternidad y crianza. 

El tipo de muestra fue no probabilística, intencional, según criterios teórico-metodológicos^38^. Para la selección de los posteos de las empresas se navegó por las cuentas Nestlé®, Sancor®, Roemmers®, Nutricia Bagó-Danone® y La Serenísima®, tanto generales como específicas, en las que publicitan sucedáneos de leche materna, y se realizó un relevamiento de los posteos realizados durante el año 2022. El criterio de selección inicial fue temporal y temático, con el propósito de identificar las estrategias de las marcas relacionadas con tres tipos de sucedáneos de leche materna: fórmulas de inicio (0/6 meses) que se comercializan y publicitan como producto etapa 1; de continuación (6/12 meses) que se comercializan y publicitan como producto etapa 2 y de crecimiento (a partir de 1 año) que se comercializan y publicitan como producto etapa 3, excluyendo otros sucedáneos de leche materna como fórmulas especiales, alimentos formulados para menores de 2 años, chupetes y biberones. Asimismo, se excluyeron cuentas generales y específicas que no tuvieron posteos referidos a esas fórmulas infantiles durante ese período. La muestra inicial quedó integrada por 168 posteos. 

Para la identificación de grupos públicos se utilizaron las palabras clave: crianza y apoyo a la crianza; madres/maternidad; embarazo; parto y puerperio. Los criterios de selección fueron que se tratase preferentemente de grupos argentinos de más de 3 mil integrantes pero, dada la dificultad inicial para relevar grupos de Argentina con esa cantidad de integrantes, se amplió el criterio y se incluyeron grupos de países de América Latina identificando su localización de acuerdo con lo informado por las personas que administran el grupo. También se utilizaron palabras claves para la identificación de posteos: fórmula; lactancia materna; leche. Los posteos incorporados a la muestra fueron los realizados durante 2022 que presentaron temas y consultas pertinentes para el estudio. Fueron excluidos posteos sobre aspectos específicos de lactancia materna sin comentarios relevantes. La muestra inicial quedó integrada por 24 posteos. Para los grupos privados, los criterios de selección fueron los mismos pero, dada la existencia de grupos argentinos, solo se incluyeron estos y se excluyeron los grupos de otros países de América Latina. Se solicitó acceso a cinco, logrando el ingreso a dos. Por razones éticas, se los menciona como Grupos 1 y 2. Logrado el acceso, se utilizó una palabra clave “fórmula” para la búsqueda de publicaciones. Los posteos incorporados a la muestra fueron publicados durante 2022. La muestra inicial quedó integrada por 116 posteos. La recolección se realizó durante los meses de diciembre de 2022 y enero de 2023. 

Realizada la selección inicial de todos los posteos, tanto de las marcas como de los grupos (n=308), se registraron y describieron los posteos en una matriz y se seleccionaron los más relevantes de acuerdo con el criterio de pertinencia y relevancia temática, contabilizando en total 142 posteos que se analizaron cualitativamente mediante la estrategia de análisis temático, a partir de categorías, definidas previamente y emergentes del propio corpus. En la Tabla 1 y la Tabla 2 se detalla la distribución de los materiales relevados y los tipos de análisis para cada caso.

**Tabla 1:** Distribución de los posteos sobre fórmulas infantiles en Facebook de las empresas, según marca/cuenta, número de seguidores y tipo de análisis. Argentina, 2022.

Nombre de la empresa	Marca/Cuenta	No. seguidores	Análisis descriptivo (n=168)	Análisis cualitativo (n=80)
Roemmers	Nutribaby	259.263	83	38
	Sancor Bebé 3	299.101	27	24
Nestlé	Baby and Me	227.203	27	7
	Nido	4.089	6	6
Nutricia Bagó-Danone	Nutrilon 4*	187.318	17	1
	Vital 4*	103.671	8	4

Fuente: Elaboración propia. *Tanto en el caso de la marca Nutrilon 4 y Vital 4 (para niños/as a partir de 3 años), ambas de Nutricia Bagó-Danone, si bien exceden la etapa 3, las cuentas fueron navegadas puesto que allí aprovechan la plataforma y publicitan Nutrilon 3 y Vital 3.

**Tabla 2:** Distribución de los posteos sobre fórmulas infantiles y lactancia materna en grupos públicos y privados sobre maternidad y crianza de Facebook, según cuentas, número de miembros y tipo de análisis. Argentina y América Latina, 2022.

Grupos	No. miembros	Análisis descriptivo (n=140)	Análisis cualitativo (n=62)
Grupos públicos			
Crianza respetuosa	304.000	15	15
Papá, Mamá & Bebes	169.800	7	7
Mi dulce espera. Embarazo. Lactancia. Maternidad	16.100	2	2
Grupos privados			
Grupo 1	3.960	62	26
Grupo 2	3.531	54	12

Fuente: Elaboración propia.

Teniendo en cuenta que los propósitos de los materiales relevados son diferentes, se definieron distintas categorías para cada actor (empresas y grupos) y diferentes herramientas para la descripción y análisis del material. En las empresas, las categorías para el abordaje fueron: *engagement* de las personas usuarias con las marcas; tácticas de promoción y venta; simbolizaciones de la maternidad/paternidad, de la crianza y de los productos; apelación a puntos de dolor *(points of pain)*; tácticas para generar cercanía, confianza y conexiones con las personas consumidoras; apelación a voces autorizadas. Para la descripción y análisis se tuvieron en cuenta referencias textuales (frases) y visuales (imágenes), además de otras herramientas de comunicación digital como *hashtags* (o etiquetas que sirven para ordenar contenidos y facilitar la conversación sobre algún tópico) y reacciones (MeGusta; Compartir y Comentar). 

En los grupos, las categorías para el abordaje del material fueron: *engagement* entre personas usuarias de grupos; simbolizaciones, sentimientos/emociones y experiencias sobre la lactancia y posicionamiento sobre el uso de las fórmulas; voces referidas. Para la descripción y análisis se tuvieron en cuenta referencias textuales (frases) y reacciones (MeGusta; Compartir y Comentar). Categorizado el material, se establecieron vínculos entre las categorías para interpretarlas, identificar sus relaciones y organizar los resultados. La presentación de los resultados se organizó a partir de la identificación de dos grandes dimensiones de análisis en función de los actores: las estrategias de marketing digital y las conversaciones entre pares para dar cuenta de sus perspectivas y luego analizar sus interacciones e identificar las huellas de las estrategias en las conversaciones. La organización y definición de las categorías se observan en la Tabla 3. 

**Tabla 3:** Matriz de procesamiento analítico de los posteos sobre fórmulas infantiles y lactancia materna seleccionados para el análisis cualitativo temático.

Dimensiones	Categorías	Definiciones
Estrategias del marketing digital	Engagement de personas usuarias con las marcas	En el idioma español, refiere a compromiso. Reacciones y referencias a conexiones, respuestas y/o experiencias de usuarios uno con el otro, con una empresa o con una marca. En el lenguaje de las redes sociales digitales, refiere al uso de las diferentes herramientas para identificar las reacciones y/o intercambios: MeGusta; Compartir o Comentar, por lo que fueron los principales indicadores.
	Tácticas de promoción y venta	Referencias (textuales, visuales y mediante hashtags) a todas las acciones explícitas que buscan promover la venta de las fórmulas, desde descuentos, cross promotion y acciones implícitas como concursos y sorteos, grupos de apoyo, contacto en tiempo real, invitaciones para visitar tienda virtual, publicación de información sobre crianza, invitaciones para saludar en días especiales, etc.
	Simbolizaciones de la maternidad/paternidad, de la crianza y de los productos	Referencias (texuales, visuales y mediante hashtags) a significados asociados a la maternidad/paternidad y a la participación de los padres en la crianza (como responsabilidad individual o compartida), referencias (textuales y visuales) a la crianza (placentera, dificultosa, trabajosa, corresponsable, otros emergentes del corpus) y a los productos (prácticos y cómodos; accesibles; de calidad; equivalentes o mejores a la leche humana; con componentes nutricionales específicos; avalados por la ciencia; recomendados por los médicos; otros emergentes del corpus).
	Apelación a puntos de dolor/puntos débiles de la lactancia	Referencias (textuales y visuales) a mensajes que apelen a puntos débiles (problemas, reales o percibidos) asociados con comportamientos infantiles comunes, como el llanto persistente y el cansancio, a partir de los cuales las marcas posicionan las fórmulas como solución. Referencias (textuales y visuales) a vinculación de los productos con la mejora del sueño; las defensas; la digestibilidad y el hambre^22^.
	Tácticas para generar cercanía, confianza y conexiones con los/as consumidores/as	Referencias (textuales y visuales) a mensajes y acciones implícitas que buscan promover la venta apelando a las emociones (culpas, angustias).
	Apelación a voces autorizadas.	Referencias (textuales y visuales) a referentes legitimados socialmente (ciencia; profesionales de la salud como médicos/pediatras; otros emergentes del corpus).
Conversación entre pares	Engagement entre personas usuarias de grupos	En el idioma español, refiere a compromiso. Reacciones y referencias a conexiones, respuestas y/o experiencias de personas usuarias unas con otras. En el lenguaje de las redes sociales digitales los indicadores para describir la conexión son las diferentes herramientas para identificar las reacciones y/o intercambios: MeGusta; Compartir o Comentar, por lo que fueron los principales indicadores.
	Simbolizaciones, sentimientos/emociones y experiencias sobre la lactancia y posicionamiento sobre el uso de las fórmulas	Referencias textuales a significados asociados a la lactancia (como responsabilidad individual o colectiva; como mandato, como derecho; otros emergentes); referencias textuales a sentimientos, emociones y experiencias (culpas, angustias, miedos, incertidumbres; otros emergentes del corpus). En relación con el posicionamiento sobre las fórmulas, referencias textuales a declaraciones sobre acuerdos o desacuerdos sobre su incorporación; motivos de incorporación e indicios de estrategias del marketing en los discursos.
	Voces referidas	Referencias textuales a referentes involucrados en la alimentación del lactante, puede incluir a profesionales de la salud e integrantes del entorno cercano (parejas, familiares, amigos/as, otros emergentes) u otros emergentes del corpus.

Fuente: Elaboración propia.

El estudio fue aprobado por el Comité Provincial de Bioética del Ministerio de Salud de la provincia de Santa Fe (Argentina), Nro. de registro 1301.

## RESULTADOS

### Las estrategias de marketing digital

#### Descripción de los posteos relevados

Del total de los materiales inicialmente relevados correspondientes a los posteos de las empresas (n=168), la mayoría (n=120) apela al lenguaje gráfico, mientras que solo 48 son posteos audiovisuales. Son variados los contenidos: desde la promoción de fórmulas centralizadas en lanzamientos de productos, promociones y puntos de venta; pasando por efemérides y llegando a posteos sobre consejería vinculada a crianza. Como parte de la estrategia elusiva, la mayoría de los anuncios se concentran en fórmulas de etapa 3, y no encontramos anuncios de etapa 1 y 2. No obstante cuando se invita a la tienda virtual, allí se encuentra el porfolio completo de productos de la compañía. 

Los posteos muestran a una madre y en ocasiones a un padre como partícipes de la crianza y recurren al lenguaje inclusivo para dirigirse a niños y niñas (“*tu hij@*”; “*l@s niñ@s*”). Representan hogares de entornos urbanos, con ingresos para adquirir los productos; y con acceso a medios y canales de compra digitales. Se construyen madres/padres preocupados por la nutrición de sus hijos e hijas y por brindarles afecto, que valoran la comodidad y la practicidad. 

Las principales destinatarias de los posteos son las madres y a ellas se dirigen con recursos como: eslóganes (“*tus instintos, nuestro apoyo*”); formas de nombrar al bebé (“*tu peque*”); extendida utilización de *hashtags* y *emojis*. Sin embargo, como se verá en las *Tácticas de promoción y venta* y en las *Simbolizaciones de la maternidad/paternidad*, muestran también la figura del padre participando de actividades vinculadas con la crianza. Además de mostrar cercanía, construyen la experiencia de la maternidad/paternidad como un desafío plagado de incertifudmbres y muestran a una madre “real” que tiene dudas y miedos, que debe ser informada, aconsejada y acompañada por profesionales médicos o por la empresa**.**

#### Engagement de personas usuarias con las marcas

En las cuentas analizadas se observó poca interacción con las personas usuarias, quienes prácticamente no destacan, comentan ni comparten posteos de las marcas. Como se observa en la Tabla 4, de los 168 posteos inicialmente relevados, 116 han recibido solo de 1 a 5 MeGusta; mientras que 41 no han recibido ningún tipo de MeGusta. Una tendencia similar se observa con otras reacciones, como comentar o compartir. 

En el primer caso, 148 posteos no han recibido ningún comentario, 19 han recibido de 1 a 5 comentarios. En el segundo caso, 136 posteos se han compartido solo de 1 a 5 veces. Cabe señalar que estas últimas herramientas revelan un mayor compromiso y se utilizan cuando hay una motivación concreta para realizar consultas y/o reclamos sobre una compra realizada o sobre algún tema tratado en los posteos. Esta poca interacción entre personas usuarias y posteos de marcas no significa que no se encuentren expuestas al marketing digital, dada la cantidad de seguidores de las cuentas (especificada en Tabla 1) y de las tácticas de captura de datos como modalidad implícita de promoción. 

Caracterizados los posteos de las empresas/marcas de la muestra inicial, se procedió a realizar un análisis cualitativo de los posteos incluidos por criterio de relevancia. 

**Tabla 4:** Distribución de posteos de Facebook de las cuentas de las empresas sobre fórmulas infantiles (n=168), según cantidad de reacciones. Argentina, 2022.

Reacciones	Posteos	%
MeGusta		
0	41	24,40
De 1 a 5	116	69,05
De 6 a 10	7	4,17
De 11 a 15	2	1,19
De 16 a 25	2	1,19
Más de 25	0	0,00
Total	168	100,00
Comentarios		
0	148	88,10
De 1 a 5	19	11,31
De 6 a 10	0	0,00
De 11 a 15	0	0,00
De 16 a 25	0	0,00
Más de 25	1	0,60
Total	168	100,00
Compartidos		
0	0	0,00
De 1 a 5	136	80,95
De 6 a 10	31	18,45
De 11 a 15	1	0,60
De 16 a 25	0	0,00
Más de 25	0	0,00
Total	168	100,00

Fuente: Elaboración propia.

#### Tácticas de promoción y venta

Las empresas utilizan una variedad de tácticas para involucrar a madres/padres a través de canales *online* y *offline*^20^^,^^26^. Si bien se observaron tácticas explícitas de venta en posteos cuya centralidad es el producto -sus beneficios y componentes, sus diferentes presentaciones- o la promoción cruzada de productos -que se comercializan estratégicamente juntos o la información sobre promociones, descuentos, puntos de venta e invitación a visitar la web- están poco extendidas, dados los límites que plantea el CICSLM. Predominan las tácticas implícitas, presentadas con lenguaje informal (*hashtags y emojis*) para generar conexión y confianza. 

Dentro de las tácticas implícitas, los posteos se dirigen a brindar información sobre salud prenatal y apoyo posnatal; información sobre nutrición, crianza y desarrollo. Como caso, ninguno de los posteos de Nestlé y su marca *Baby and Me* muestran el producto que buscan comercializar, pero ofrecen información de interés; asesoría; acompañamiento para vivir la experiencia de la maternidad de manera placentera. En la mayoría de los posteos se ofrece la posibilidad del registro y el contacto, a través de invitaciones a visitar la web de la empresa o la tienda virtual o con la presencia del chat *on line*. 

Otra forma de registrar datos, la encontramos en la invitación al relato de experiencias sobre maternidad o al registro de saludos en días especiales. En cualquier caso, en los posteos donde es evidente la promoción de fórmulas suele aparecer, aunque sin sistematicidad, la leyenda: “*No es sucedáneo de leche materna. Complementa la alimentación de niños de corta edad*”. También se sugiere consultar con el médico de confianza, como garantía para el uso de fórmula. En los posteos donde ese fin último aparece velado a través de una propuesta de asesoría también suelen aparecer las leyendas: “*No dudes en consultar a tu pediatra o médico de confianza*”.

#### Simbolizaciones de la maternidad/paternidad, de la crianza y de los productos

**Figura 1:**
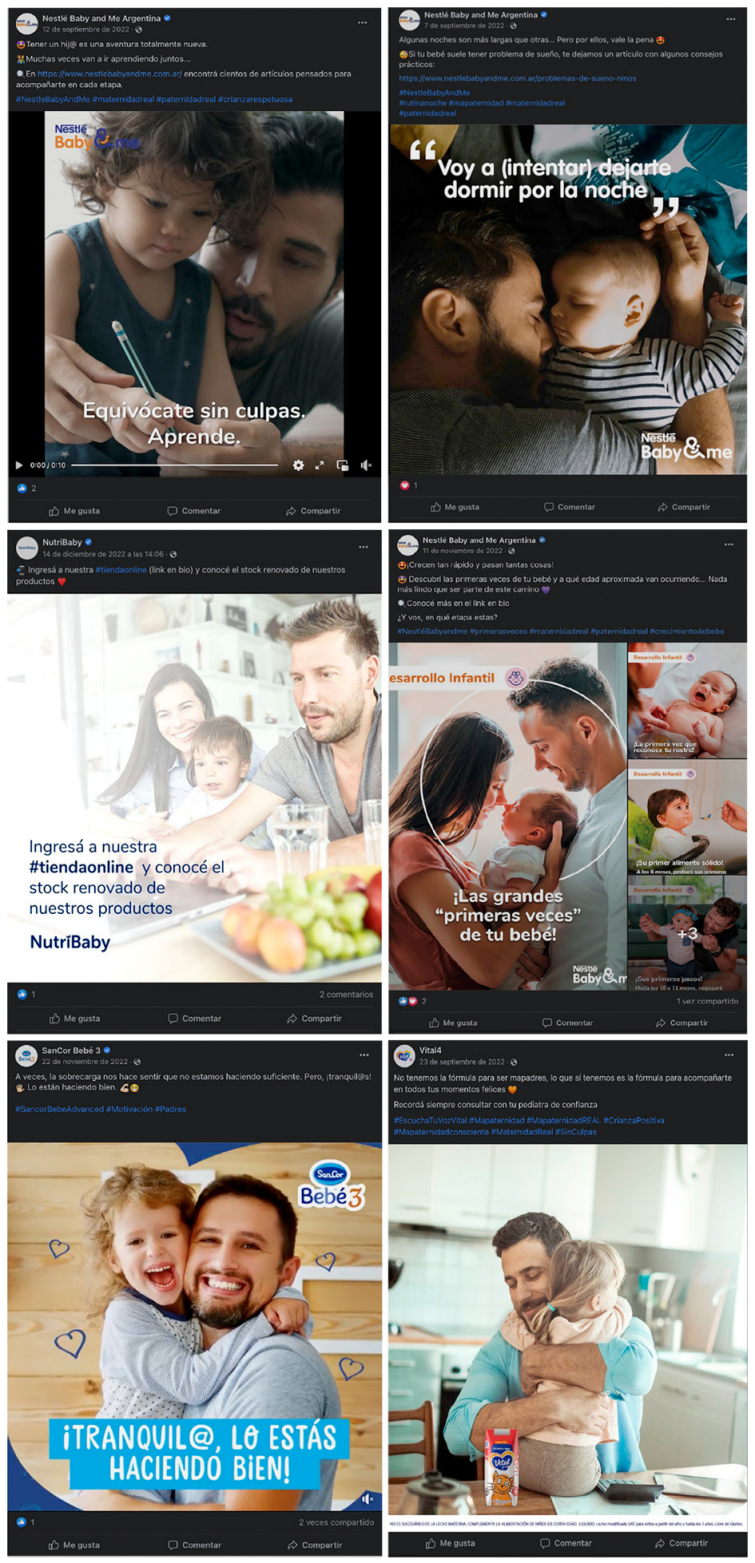
Simbolizaciones de la participación masculina en la crianza en un grupo de posteos en Facebook de Nestlé Baby and Me Argentina, Vital 4 y SanCor Bebé 3. Argentina, 2022.

**Figura 2:**
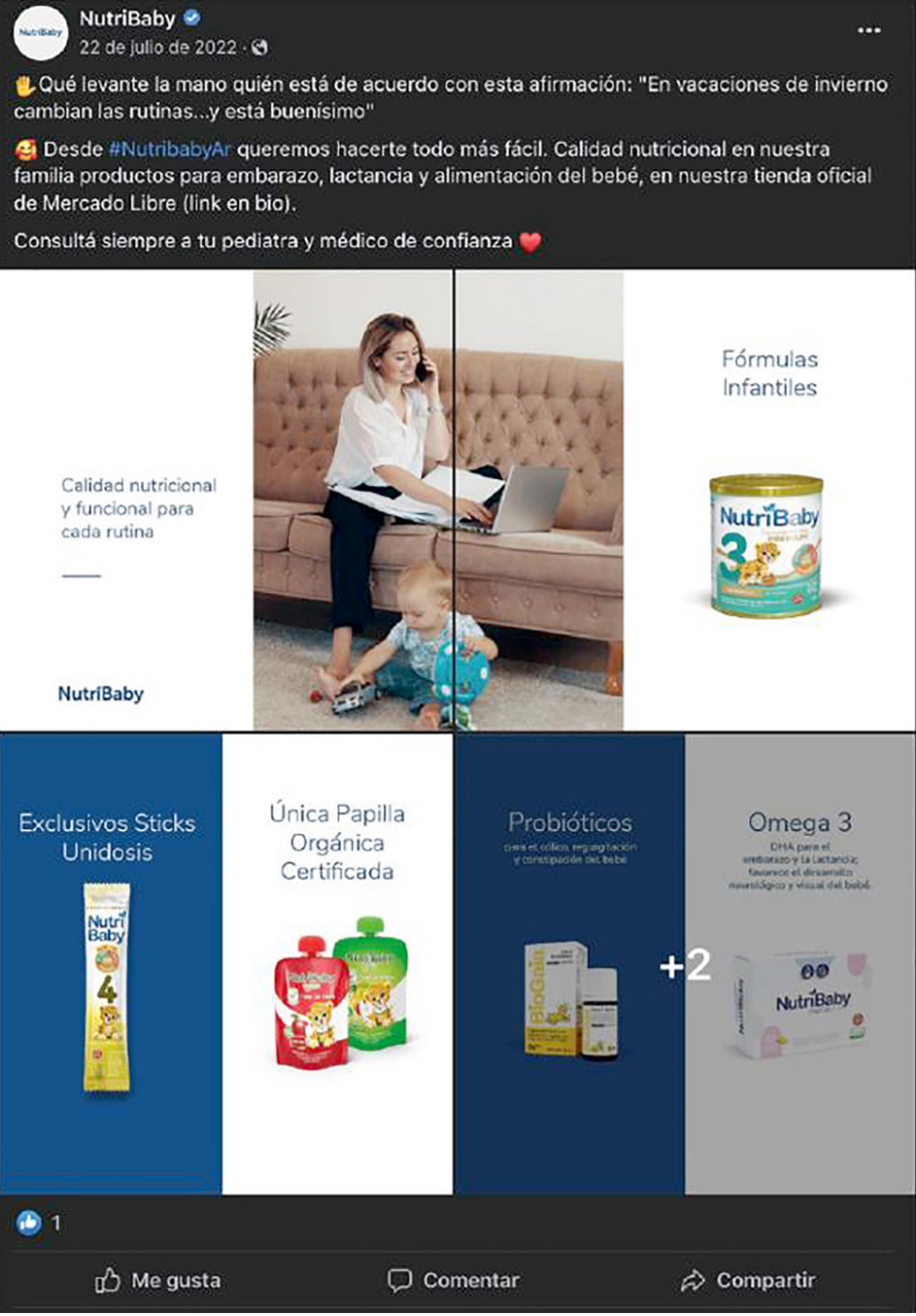
Posteo de Nutribaby, 22 de julio de 2022.

Las estrategias del marketing digital sintetizan valores de maternidad/paternidad posmodernos a través de la participación masculina en la crianza^39^^)^ (Figura 1); el acceso de medios digitales para la búsqueda de información, asesoría y compra; la búsqueda de practicidad y comodidad acorde con un modelo de maternidad y paternidad “real” y un modo de vida urbano (Figura 1 y Figura 2). 

Fuente: Cuentas de Facebook Nestlé Baby and Me Argentina, Vital 4 y SanCor Bebé 3

Fuente: Cuenta de Facebook Nutribaby

Representan la felicidad de bebés y familias, que realizan actividades juntos con afecto. Puede sintetizarse en “*la crianza se disfruta*”, aprovechando para vender experiencias de maternidad/paternidad corresponsables del cuidado, que se observan a través de imagénes en las que se muestra al padre participando de diferentes actividades de la crianza (como gestionar el sueño, enseñar a caminar, dar de comer, abrazar y comprar fórmulas) (Figura 1). 

Suelen utilizar frases de inicio de posteos como: “*Bienvenid@ a la mapaternidad* […] *Nuestra recomendación es no hacer cosas o tomar decisiones con el fin de complacer al resto porque cada mapaternidad es única y nadie conoce la tuya mejor que vos*” (posteo de Vital4, del 23 de enero de 2022) y de hashtags como: #MapaternidadREAL, #Mapaternidadconsciente (en posteos de Vital 4, 23 de septiembre de 2022); #paternidadreal (en posteo Nestlé Baby and Me Argentina, 7 de septiembre de 2022); #padres (en posteos de SanCor Bebé 3, 22 de noviembre de 2022). Adicionalmente, y como han señalado otras investigaciones^2^, las fórmulas permiten que las mujeres estén libres de los condicionamientos biológicos que implican una responsabilidad exclusiva en la alimentación infantil y que las parejas tengan un papel en el cuidado infantil. 

Al igual que otras investigaciones sobre promoción de sucedáneos de leche materna^6^ en las que evidenciaron mensajes que destacan practicidad de los productos, los comparan con la leche humana; destacan sus propiedades nutricionales y saludables, en nuestro análisis también encontramos que los productos se presentan como de máxima calidad, con componentes equivalentes o superiores a los de la leche humana. En un posteo de Nutribaby se afirma que la línea de fórmulas infantiles “*está desarrollada bajo una estricta mirada farmacéutica, garantizando los más altos estándares de calidad*”. Este enfoque se aprovecha del deseo de dar lo mejor para hijos e hijas y justifica que paguen precios mayores. 

Como lo viene haciendo la publicidad de productos para alimentación infantil^23^, los posteos se centran en que los productos contribuyen al desarrollo cognitivo; al fortalecimiento del sistema inmunológico y a la optimización del sistema digestivo. Un posteo de Sancor Bebé 3, afirma:

*Tenemos la fórmula para que tu peque reciba todos los nutrientes que necesita durante una etapa fundamental de su vida. Sancor Bebé Advanced 3 contiene: MFGM + Prebióticos: fortalecen el Sistema Inmune. Neuronutrientes DHA + MFGM: favorecen el Desarrollo Cognitivo. Prebióticos GOS + PDX: que ayudan a la Digestibilidad. Sancor Bebé Advanced es Innovación en Nutrición.*

Por último, al igual que la investigación de Vallone^6^, los productos se promocionan como cómodos y prácticos. A través de diferentes presentaciones (Figura 2) se pueden preparar en cualquier lugar, respondiendo a uno de los beneficios de la lactancia, pero sumando la autonomía de la madre. 

#### Apelación a puntos de dolor

El marketing se nutre de puntos débiles de la lactancia que generan angustias y ansiedades en las madres y pueden llevarlas a creer que necesitan fórmulas para afrontar problemas -reales o percibidos- vinculados con comportamientos comunes de los bebés, como llanto persistente, corta duración del sueño, cansancio e irritabilidad^3^^,^^22^. Estos problemas, en consonancia con la responsabilidad asignada a la madre para el cuidado^15^, se asocian con la calidad y cantidad de la leche para satisfacer el hambre^3^. Como la ganancia de peso del bebé aparece como tema central, funciona como indicador de desempeño exitoso de una buena maternidad^9^ socavando la confianza de la madre en la lactancia y aumentando su sentimiento de inseguridad y autoeficacia. 

**Figura 3:**
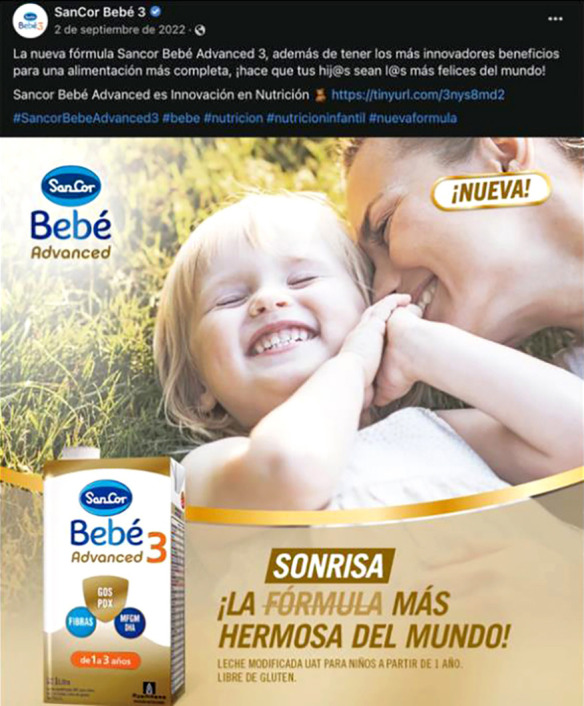
Posteo de Sancor Bebé 3, 2 de septiembre de 2022.

Otra de las preocupaciones son los cólicos, también aprovechados por los mensajes de marketing^3^. En algunos posteos se enfatiza cómo los productos “*ayudan a la digestibilidad*”, como el caso de Sancor Bebé 3. Por ello, hay tres ideas fuerza en los productos: ofrecen defensas (para que no enferme); ayudan a la digestibilidad (sortean mejor los cólicos, cuestionando la idea de que las fórmulas producen estreñimiento); contribuyen al crecimiento (marcado por el peso). Así, las fórmulas infantiles se posicionan como una alternativa, en algunos casos, igual o mejor a la leche materna, gracias a la innovación científica que resuelve problemas^2^ y que sintetizan la idea de crecimiento sano, fuerte y feliz (Figura 3) con una clara tendencia a la medicalización de los comportamientos infantiles. 

Fuente: Cuenta de Facebook Sancor Bebé 3.

**Figura 4:**
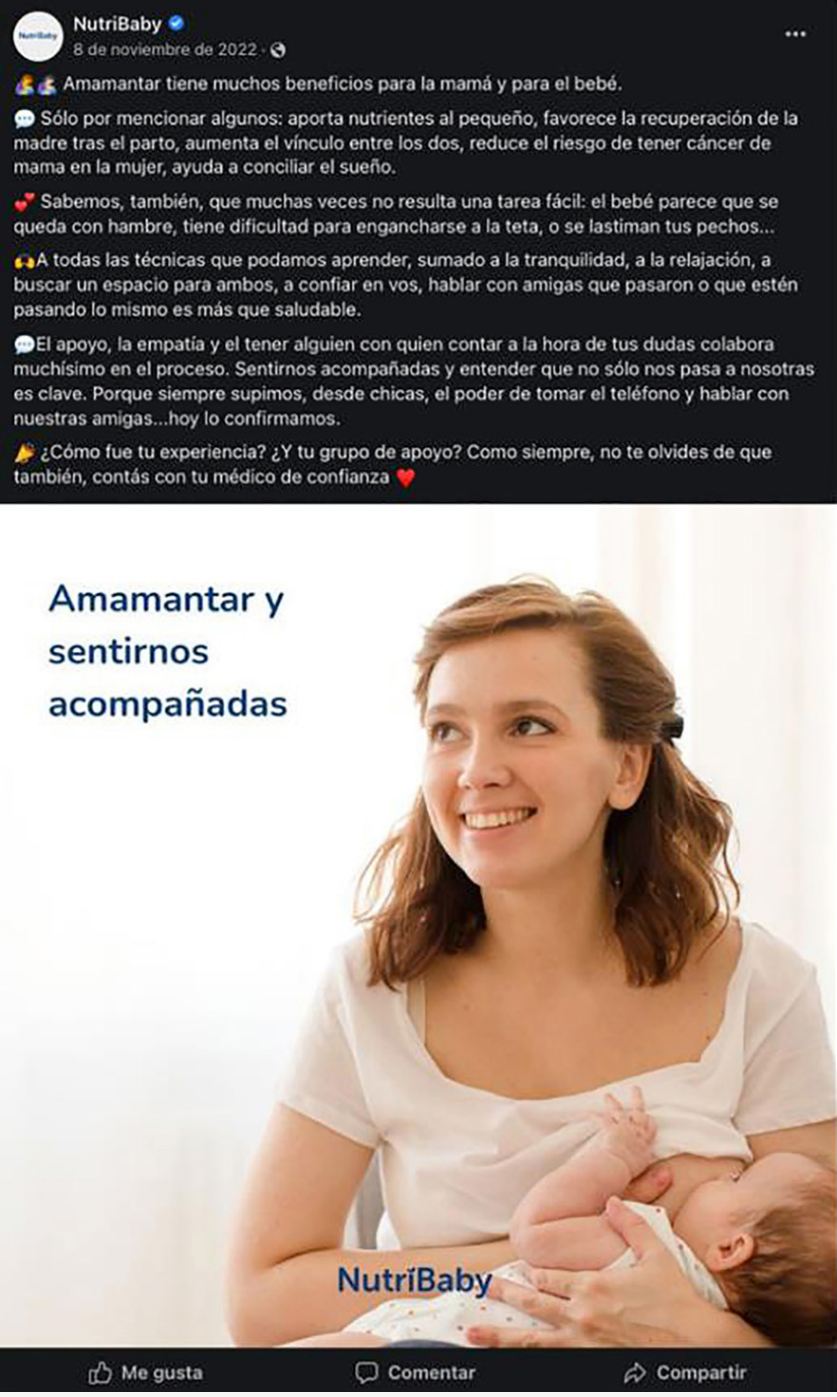
Posteo de Nutribaby, 8 de noviembre de 2022.

Es recurrente la línea argumental sobre los beneficios, pero también dificultades de la lactancia materna: “*Sabemos también que muchas veces no resulta tarea fácil: el bebé parece que se queda con hambre, tiene dificultad para engancharse a la teta, o se lastiman tus pechos*…”. Refuerza la idea del apoyo y la empatía y la presencia del médico de confianza. Invita a contar la experiencia allí mismo. Podría ser un posteo sobre lactancia materna, si no fuera porque se trata de una empresa que vende fórmulas infantiles y, aunque no muestra el producto, presenta una táctica implícita de comercialización extendida que recupera los puntos débiles de la lactancia^21^ y la necesidad de apoyo que buscan las madres para sostenerla^37^ (Figura 4).

Fuente: Cuenta de Facebook Nutribaby.

#### Tácticas para generar cercanía, confianza y conexiones con las personas consumidoras

Además de afirmar que sus productos pueden resolver problemas comunes de los bebés, las marcas se posicionan como confiables consejeras que acompañan su crecimiento, trabajando sobre incertidumbres, miedos y angustias de madres/padres (Figura 1 y Figura 4). Recuperando la centralidad de las emociones en las experiencias de la lactancia^16^, no juzgan, apoyan y emplean tácticas para generar confianza nutriéndose de las emociones que se ponen en juego en la lactancia^21^: “*Siempre cerca tuyo*”, reza un posteo de Nutribaby. Al igual que otros investigadores^2^ que destacan que el marketing busca generar confianza con las mujeres y dar la impresión de que la industria de fórmulas está de su lado, en nuestro análisis hemos visto que la erradicación del juicio es un enfoque central de la comercialización. Al presentarse como amigables, mitigan la culpa en torno al uso de los productos, sugiriendo que el uso de fórmulas forma parte de la preocupación por la alimentación del bebé. “*Tus instintos, nuestro apoyo*”, reza el eslogan de *Baby and Me*, de Nestlé. O “*equivocate sin culpa*”, reza otro cierre de posteo de la misma marca. O, en Vital 3, afirma: “*No tenemos la fórmula para ser mapadres, lo que sí tenemos es la fórmula para acompañarte en todos tus momentos felices*”. 

#### Apelación a voces autorizadas

La apelación a la ciencia como fundamento de calidad está muy extendida en posteos centrados en los beneficios del producto^2^^,^^8^^,^^10^^,^^21^^,^^29^. Menciones a la innovación científica para la mejora del producto y de sus componentes específicos, representados en siglas en inglés, como MFGM y HMO (membrana del glóbulo graso y oligosacáridos de la leche materna, respectivamente); prebióticos; simbióticos; neuronutrientes; microbiota; términos desconocidos para madres/padres pero que asumen que son avalados por laboratorios reconocidos o por la trayectoria de las empresas y son similares a los componentes de la leche humana por lo que deben ser buenos y equivalente o superiores a la leche humana. En un posteo de Sancor Bebé 3, se afirma: “*El motor de nuestra investigación fue, es y será aportar lo mejor a tus hijos en su etapa más importante de crecimiento y desarrollo*”. 

Si la ciencia es el fundamento de la calidad gracias a la innovación, las voces autorizadas para recomendar su uso son las y los profesionales de la medicina. En la mayoría de los posteos en los que se ofrecen consejos o información sobre nutrición y crianza o se promociona el producto, se sugiere consultarlos porque apoyan, aconsejan y acompañan (Figura 1, Figura 2 y Figura 4). Pero porque, especialmente, son quienes indican la fórmula. Tanto es así que también se los saluda en su día, construyéndolos en ese caso en particular como destinatarios directos del mensaje, pero en todos se evidencia la alianza y su uso como recurso del marketing por erigirse en intermediarios protagónicos^2^^,^^6^. 

### Las conversaciones entre pares

#### Descripción de los posteos relevados

Las redes sociales, además de ser plataformas de promoción, son utilizadas por las personas usuarias para el intercambio de conocimientos y experiencias vinculadas con la maternidad y la crianza^16^^,^^37^. En los grupos se comparten posturas, comportamientos y actitudes, erigiéndose en escenarios para el estudio de la interacción entre usuarios. Los grupos públicos seleccionados ofrecen acompañamiento y apoyo: “*este grupo es para sentirnos acompañadas y acompañados en esta dura tarea que es la crianza respetuosa y responsable*”; “*conflictos que no se pueden hablar con familiares y amigos*”. Los propósitos de los grupos privados son similares: “*compartir información*”; “*hacer preguntas, compartir experiencias, opiniones*”. Ambos reflejan que las madres se guían por recomendaciones de otra par y las complementan con las de las y los profesionales^40^. En sus reglas de funcionamiento se prohíbe la venta, comercialización e intercambio de servicios y productos. En uno de ellos se indica, entre otras normas: “*Respetar indicaciones de la OMS*” y “*Ante cualquier emergencia siempre ir al médico, podemos dar consejos, pero siempre lo mejor es consultar con un profesional*”. 

Todas las publicaciones y comentarios corresponden a madres y los grupos están integrados por ellas. No se observa presencia ni referencia a padres y abuelas o abuelos. Las publicaciones suelen comenzar “*Hola grupo*” u “*Hola chicas*”, y la conversación es informal y distendida, cálida y afectuosa, especialmente cuando se responde una consulta de alguien que está triste o abatida. 

El tipo de publicación predominante es escrita y sin ilustraciones, con excepción de publicaciones en las que se comparten fotos de envases de fórmulas sobre los que se hace alguna consulta acerca de su calidad, o bien imágenes alusivas a la lactancia como práctica idealizada. Es frecuente el uso de *emojis* sustituyendo palabras. El tipo predominante de contenido es la consulta (95 publicaciones sobre 116 de grupos privados, y 21 sobre 24 de grupos públicos). Estas son consultas prácticas (qué marca de fórmula comprar, cómo prepararla, qué dosis corresponde según edad/peso del niño, cómo conservarla). Aunque en menor medida, es posible encontrar pedidos de consejo u orientación para afrontar problemas vinculados con la lactancia (disminución de la producción de leche; incorporación de leche de fórmula y su impacto en la producción; destete; cólicos, constipación o diarreas en el bebé; rechazo de la teta y/o biberón; llanto reiterado, etc.) y a situaciones de crisis de crecimiento asociadas con el no aumento esperable de peso. 

Solo se registraron algunos casos de publicaciones consistentes en declaraciones en las que se narra una experiencia aleccionadora, se expresa un estado de ánimo o sentimiento, y/o se declara posicionamiento sobre un asunto importante o polémico para el grupo: una madre expresa su necesidad de hacer “catarsis” por la culpa y la frustración que le generó recurrir a la fórmula; otra comparte su decepción y la consecuente decisión de cambiar de pediatra; una madre comunica que se siente orgullosa por haber sostenido durante dos años y cinco meses la lactancia, habiendo prescindido del uso de fórmula, a pesar de la falta de información y acompañamiento; y otra publica un texto en el que compara los beneficios de la leche materna con la utilización de fórmulas, destacando los beneficios de la primera sobre las segundas. 

#### Engagement entre personas usuarias de grupos

De la variedad de publicaciones, los pedidos de consejo u orientación sobre problemas o puntos de dolor^26^, por un lado, y los sentimientos de tristeza, culpa y frustración, por otro, son los que suscitan más interacciones consistentes en expresiones de identificación, recomendaciones o palabras de aliento. Por la dinámica de los grupos, las publicaciones tienen más comentarios que reacciones (MeGusta). En cambio, las declaraciones que comparten textos informativos y no aluden a experiencias personales ni emociones, tienen un alto número de reacciones, pero no reciben comentarios. Las publicaciones con profunda implicación personal suelen provocar mayor cantidad de interacciones y comentarios por el compromiso de pares que implica la integración de estos grupos^31^. 

#### Simbolizaciones, sentimientos/emociones y experiencias sobre la lactancia y posicionamiento sobre el uso de las fórmulas

Al igual que otras investigaciones sobre lactancia^13^^,^^14^, el análisis de posteos y comentarios evidenció que las mujeres sienten que deben cumplir con el mandato social, cultural y sanitario de amamantar y que, cuando no lo hacen, se sienten culpables, frustradas o fracasadas; juzgadas como malas madres desde una concepción de la maternidad caracterizada por la entrega y el sacrificio (de tiempo, de abnegación, de esfuerzo, de voluntad, de horas de sueño, de alimento de calidad) que debe anteponer la salud y el bienestar del niño o de la niña a sus necesidades, preferencias o conveniencias^11^^,^^17^. 

No obstante, y de acuerdo con investigaciones que marcan otras concepciones sobre la maternidad^9^^,^^11^, la lactancia también es presentada como una elección de la madre en la que no deben interferir opiniones de terceros. Si amamantar no le resulta placentero, debe priorizar su propia felicidad para garantizar un buen vínculo con su hijo, más allá de la lactancia. El siguiente fragmento conjuga el mandato de la lactancia exclusiva, los sentimientos de culpa y frustración derivados de su incumplimiento con la elección de la fórmula, pero también, como lo muestran otras investigaciones^9^, la resignificación de la asociación entre lactancia, bebé sano y “buena maternidad”:

*No SOS peor madre por dar fórmula, el “oro puro” que le podés dar a tu hijo SOS vos, una mamá fuerte y feliz* […] *Yo lo intenté y no pude ser LME y no soy ni peor ni mejor madre* […]. *Así que* […] *no sientas culpa* […]. *Hay mil niños sanos y fuertes criados a fórmula* (usuaria grupo privado).

En cuanto a las huellas del discurso publicitario, la característica de un niño feliz e inteligente se asocia a la buena alimentación y aparece en los argumentos de venta^23^. No obstante, la fórmula aparece como segunda opción aceptada con resignación cuando la lactancia no puede practicarse. Quizá por principios de los grupos, no se registraron conversaciones en las que mujeres declaren abiertamente elegir fórmula pudiendo amamantar exclusivamente, ni siquiera como complemento para aliviar la demanda que supone sostener la lactancia y, fundamentalmente, exclusiva. 

Cuando las mujeres mencionan que usan fórmula, lo justifican más allá de su deseo, conveniencia o comodidad (“*no tengo leche suficiente*”, “*mi bebé se queda con hambre*”, “*me lo indicó el pediatra porque el bebé no aumentaba de peso*”), y lo experimentan con tensiones y contradicciones^9^. Por tanto, la lactancia se equipara con buena maternidad cuando se considera que el bebé está sano y feliz, pero si un bebé amamantado no está ganando peso o está intranquilo, por los factores que sea (personales o contextuales), constituye una razón para acudir a la fórmula^13^, puesto que el crecimiento saludable y las necesidades del bebé son primordiales. Pero la incorporación de fórmula y el pasaje a la lactancia mixta a pedido de la madre aparece habilitada cuando se pone en riesgo su bienestar físico (dolor o desgaste)^13^^,^^14^ o su felicidad o por razones laborales o médicas. 

En las conversaciones no aparecen menciones a contactos con empresas. Tampoco referencias a tácticas de marketing, con excepción de una madre que comenta que había recibido una muestra gratis de una marca y de otra madre que criticaba el marketing digital y sus argumentos de venta:

…*las fórmulas tienen grandes cantidades de azúcar, no te dejes engañar por publicidad engañosa, que sirven para el DHA y m4m4m4*… (usuaria grupo público)

Sí se registraron casos en los que se hacen consultas al grupo y se comparten imágenes del producto que indican el conocimiento de páginas o e-shop de las marcas. Como parte integrante del aprendizaje implicado en la experiencia de la maternidad^37^, las mujeres están familiarizadas con las fórmulas, las compran y han probado diferentes hasta dar con la marca de su preferencia, ya sea como complemento de la lactancia o uso exclusivo. En consonancia con otras investigaciones^13^, las mujeres explican que las fórmulas fueron incorporadas por indicación del pediatra, quien aconseja una marca específica de inicio cuando el o la bebé pierde peso o no cumple con los estándares de peso esperables para su edad. Como también evidenciaron otras investigaciones^41^, a propósito de opiniones profesionales sobre lactancias prolongadas, se observaron conversaciones referidas a “*pediatras desactualizados*” que desconocen los parámetros para determinar cuál es el peso esperable en niños y niñas con lactancia exclusiva. 

Luego de iniciar el uso de fórmula, las madres consultan con pares y prueban otras marcas que eligen según precio, recomendación de amigas o miembros del grupo, o de acuerdo con cómo su bebé las digiere y/o cuánto le agrada. En lo que respecta a las fórmulas para niños y miñas mayores a un año, en la mayoría de las conversaciones se observa acuerdo con la idea de que luego del año de edad corresponde el uso de leche de vaca, pasteurizada y entera, y que no es conveniente el uso de fórmula por su exceso de azúcares. Sin embargo, se destacan comentarios de madres “*ultra informadas*” que apelan al conocimiento y lenguaje técnico de los expertos para justificar sus decisiones^11^ y contradecir esta idea, como una madre que comparte una captura de la información nutricional del envase del producto Nutrilon como prueba de que no contiene azúcar. 

#### Voces referidas

En las conversaciones entre madres pares, no aparecen padres ni abuelos o abuelas como actores con peso en decisiones y gestiones relativas a la lactancia. Sí, el médico o la médica. Como evidenciaron otras investigaciones^25^^,^^27^, el o la pediatra es determinante de la incorporación de la fórmula y es habitual que prescriba una marca específica. La indicación está asociada al logro del peso “saludable”^14^. Con la experiencia del suministro de la fórmula y el consejo de otras madres pares, la madre suele cambiar de marca, sin consultar o informar a su pediatra de este cambio. Así cuando la madre duda o gana experiencia, esta palabra es puesta a prueba y juzgada en el grupo de pares que puede aconsejar la necesidad de cambiar de pediatra por alguien más “*empático*” o “*actualizado*”. La convicción de que las y los profesionales están desactualizados en temas de la salud infantil hace que las madres asuman una actitud defensiva, de confrontación o de cambio^22^. En tanto voces de autoridad, aparecen señaladas como responsables de situaciones de vulneración de derechos, por no brindar asesoramiento adecuado. 

Otras investigaciones también han indicado a la figura de la puericultora^39^, indicada para madres con problemas para sostener la lactancia. Se trata de mujeres asesoras, mejor preparadas, más accesibles y cercanas que el o la pediatra. Es frecuente que en el grupo se compartan contactos para agendar turnos/consultas o enlaces a contenidos relevantes en las cuentas de puericultoras. Varias de ellas integran el grupo, ofrecen consejos y agregan su contacto para consultas privadas, autorizadas por las administradoras.

Más allá de estas figuras, la experiencia de las madres pares aparece como referencia y complemento del saber experto, lo que explica la necesidad de participar de estos grupos^41^ en los que se suele encontrar apoyo y acompañamiento frente a los desafíos para sostener la lactancia materna^37^. 

## DISCUSIONES

La promoción de fórmulas comerciales infantiles no es nueva, pero con el desarrollo del entorno digital sus tácticas se han sofisticado para eludir el CICSLM y perfilar clientes, segmentar audiencias y focalizar estrategias de marketing^2^^,^^21^^,^^31^^,^^42^. Aunque difícilmente distinguible, observamos que el contenido promocional es omnipresente. En sintonía con investigaciones previas^21^, observamos diferentes tácticas para promover las ventas, exponiendo a las familias con niños y niñas menores de dos años, especialmente a las madres, al marketing digital. Las cuentas de las empresas de fórmula rara vez refieren al producto en sus contenidos sobre crianza y desarrollo. Cuando las empresas hablan de productos, priorizan las promociones de marca y privilegian los de la etapa 3 y 4 mediante promoción cruzada. 

Asimismo, las estrategias publicitarias asocian los productos con determinados significados^34^. Cuando la identidad corporativa aparece asociada a un laboratorio las estrategias adoptan los parámetros de la publicidad farmacéutica, en la que la información biomédica y la innovación científica estructuran el mensaje y presentan la fórmula como sustituto equivalente o superador de la leche humana^8^^,^^29^, en el marco de construcciones sociales de una maternidad/paternidad preocupada por la alimentación de sus hijos e hijas. Las estrategias se nutren de los desafíos (laborales, económicos, sociales y emocionales) implicados en la lactancia, de los diversos contextos de vida de las mujeres y de concepciones de “buena maternidad” asociadas con la preocupación primordial por el crecimiento de hijos e hijas, a la vez que problematizan la alimentación infantil como responsabilidad individual exclusiva de la madre^2^^,^^17^ y el vínculo causal entre “buena madre” y lactancia^9^. 

Las usuarias tienen comportamientos diferenciados según se trate de cuentas de empresas o grupos de pares: prácticamente no “likean”, no comentan ni comparten contenidos de empresas, pero las siguen y están al tanto de sus publicaciones, dada la cantidad de seguidores de las cuentas y los contenidos de posteos y comentarios realizados en los grupos. Por lo que sus datos personales son capturados para la segmentación y focalización, estando expuestas a las tácticas de marketing digital. Las madres conocen, usan y prueban diferentes marcas de fórmulas. No se registra en sus discursos la huella del discurso publicitario, el uso del lenguaje o la mención de atributos de los productos tal como son referidos en los posteos de las empresas, aunque sí se observa un correlato entre atributos del producto publicitados con resignificaciones de la concepción de la “buena” maternidad, y con puntos de dolor, aspiraciones y motivaciones referidas en los grupos, lo que debería explorarse con otras técnicas cualitativas para comprender sus interrelaciones. 

Si bien las madres son el objetivo del marketing, al igual que otras investigaciones^2^^,^^18^^,^^19^^,^^20^^,^^26^, encontramos que las y los profesionales de la salud son también figuras protagónicas, objetivos y a la vez recursos del marketing. Como estrategia generalizada, las empresas construyen a médicos y médicas como aliados e invierten en ellos, en sus asociaciones y en sus revistas científicas^6^ para generar familiaridad, credibilidad y compromiso de reciprocidad^2^. 

La indicación profesional de fórmulas para bebés que no cumplen con estándares de peso recomendado es frecuente en posteos y comentarios de los grupos. Como señalaron otras investigaciones^3^ el incremento del peso genera una importante presión sobre las madres como responsables del crecimiento, lo que termina justificando la incorporación de la fórmula. En dichas situaciones, la narrativa pro lactancia materna exclusiva opera como un mandato experimentado de manera individual, que genera sentimientos de culpa, frustración y angustia, que luego son aprovechados por el marketing digital^2^ y que hemos observado en nuestro estudio. Enmarcar la lactancia como una cuestión de responsabilidad individual y, en particular, exclusiva de las mujeres^2^^,^^13^ alienta a estas a culpabilizarse por no amamantar, por lo que es fundamental considerar las diversas concepciones de maternidad, así como diversos valores atribuidos a las prácticas de alimentación y sus consecuencias en la salud de niños y niñas. 

Concluimos que para contrarrestar los argumentos de venta presente en los mensajes comerciales sobre el papel de las fórmulas en la ganancia del peso como indicador de éxito de crecimiento sano y fuerte y evitar la medicalización de los comportamientos esperados de las y los bebés^27^ es fundamental problematizar los significados asociados con el peso saludable en lactantes y revisar las prácticas relativas a sus controles de salud, poniendo atención en la lactancia^10^^)^ y su relación con el incremento de peso en las primeras semanas de vida. Asimismo, es fundamental sensibilizar a las y los profesionales sobre la alimentación de niñas y niños pequeños y sobre cómo informar, recuperando las diversas experiencias de la maternidad^37^. 

Adicionalmente, dado que los mensajes comerciales enfatizan otros puntos débiles de la lactancia asociados con los comportamientos esperados de las y los bebés (como llanto persistente, corta duración del sueño, cansancio, irritabilidad) y con el cansancio y/o dolor de la madre, las fórmulas se presentan como capaces de intervenir eficazmente en los desafíos implicados, ofreciendo autonomía, confianza y apoyo y trabajando sobre los aspectos sociales y emocionales. Por esta razón, es importante capacitar a profesionales sobre la lactancia desde una perspectiva de género. Promover una atención culturalmente apropiada y centrada en las mujeres y ubicar a la lactancia como responsabilidad colectiva dentro del trabajo de cuidados compartidos^10^ y no como responsabilidad individual de la mujer^2^ podría revertir la atención materna excesivamente medicalizada en perjuicio de la lactancia; favorecer la orientación sobre los aspectos sociales y emocionales involucrados en la salud de las mujeres, además de los médico-nutricionales asociados con la alimentación de las y los bebés.

Finalmente, las autoridades deben desarrollar regulaciones para los entornos digitales e implementar programas educativos para informar sobre prácticas comerciales desleales o ilegales en la web. Asimismo, es necesario proteger la integridad de las ciencias y la medicina, de tal manera que las y los profesionales y sus asociaciones adopten comportamientos éticos y aboguen por políticas para intervenir en los conflictos entre los intereses corporativos y los de la salud pública.
